# Treatment of symptomatic hyponatremia with hypertonic saline: a real-life observational study

**DOI:** 10.1530/EJE-20-1207

**Published:** 2021-02-25

**Authors:** Irina Chifu, Amelie Gerstl, Björn Lengenfelder, Dominik Schmitt, Nils Nagler, Martin Fassnacht, Dirk Weismann

**Affiliations:** 1 Division of Endocrinology and Diabetology, Department of Internal Medicine I, University Hospital of Wuerzburg, University of Wuerzburg, Wuerzburg, Germany; 2 Intensive Care Unit, Department of Internal Medicine I, University Hospital of Wuerzburg, University of Wuerzburg, Wuerzburg, Germany; 3 Division of Cardiology, Department of Internal Medicine I, University Hospital of Wuerzburg, University of Wuerzburg, Wuerzburg, Germany

## Abstract

**Objective:**

Treatment of symptomatic hyponatremia is not well established. The European guidelines recommend bolus-wise administration of 150 mL of 3% hypertonic saline. This recommendation is, however, based on low level of evidence.

**Design:**

Observational study.

**Methods:**

Sixty-two consecutive hyponatremic patients admitted to the emergency department or intensive care unit of the University Hospital Wuerzburg were divided in subgroups according to treatment (150 mL bolus of 3% hypertonic saline or conventional treatment) and symptom severity. Treatment target was defined as an increase in serum sodium by 5–10 mEq/L within first 24 h and maximum 8 mEq/L during subsequent 24 h.

**Results:**

Thirty-three out of sixty-two patients (53%) were presented with moderate symptoms and 29/62 (47%) with severe symptoms. Thirty-six were treated with hypertonic saline and 26 conventionally. In the hypertonic saline group, serum sodium increased from 116 ± 7 to 123 ± 6 (24 h) and 127 ± 6 mEq/L (48 h) and from 121 ± 6 to 126 ± 5 and 129 ± 4 mEq/L in the conventional group, respectively. Overcorrection at 24 h occurred more frequent in patients with severe symptoms than with moderate symptoms (38% vs 6%, *P *< 0.05). Diuresis correlated positively with the degree of sodium overcorrection at 24 h (r = 0.6, *P* < 0.01). Conventional therapies exposed patients to higher degrees of sodium fluctuations and an increased risk for insufficient sodium correction at 24 h compared to hypertonic saline (RR: 2.8, 95% CI: 1.4–5.5).

**Conclusion:**

Sodium increase was more constant with hypertonic saline, but overcorrection rate was high, especially in severely symptomatic patients. Reducing bolus-volume and reevaluation before repeating bolus infusion might prevent overcorrection. Symptoms caused by hypovolemia can be misinterpreted as severely symptomatic hyponatremia and diuresis should be monitored.

## Background

Hyponatremia (HN) is the most common electrolyte disturbance in hospitalized patients and is, at the same time, subject to considerable uncertainties in understanding and clinical decision making (1). This is in contrast to the well-established treatment of hypokalemia, where the pathophysiology is also poorly understood, but treatment initiation and control occurs without significant effort in clinical routine (2). Particularly in the case of emergencies, treatment of HN is complicated by the fact that an easy, reliable and validated treatment strategy is not established.

Two major complications occur in HN: first, fatal cerebral edema due to a rapid decrease of serum osmolality and secondly, the osmotic demyelination syndrome as a result of too fast increase in serum osmolality (3, 4, 5, 6). In fact, these complications mark the extremes of under- or overtreatment. The European practice guidelines for the treatment of HN came up with a feasible approach to prevent edema and overcorrection, respectively (7). First, a symptom-based decision making is introduced and secondly, a bolus-based treatment approach with 3% hypertonic saline (3% NaCl) is recommended. Focusing on the clinical presentation, the practice guidelines recommend classifying patients based on symptoms related to cerebral edema and decide about emergency interventions accordingly. Every patient with new onset (acute) or worsening (chronic) HN suffers from cerebral edema to a certain degree, and, importantly, the severity of the edema is reflected by symptoms of cerebral pressure ranging from asymptomatic to coma (7, 8, 9) (see also Table 1). Prevention of overcorrection, instead, is achieved by a bolus-wise administration of 3% NaCl in combination with carefully controlling serum sodium (sNa) to comply with the recommended range of daily increase (8, 9). While both recommendations are physiologically very well justified, they are both, at the best, supported by very low evidence only (7). Moreover, practice shows that sodium shifts beyond the recommended limits are frequent (1).

**Table 1:** Classification of hyponatremia according to time-to-onset and symptom severity (7).

Criteria	Values
Biochemical severity*	
Mild	130–135
Moderate	125–130
Profound	<125
Time-to-onset, hours	
Acute	<48
Chronic	≥48
Severity of clinical presentation^†^	
Moderately severe	Headache, confusion, nausea
Severe	Emesis, cardiorespiratory distress, somnolence/coma (GCS ≤ 8), seizures

*Measured as serum sodium (mEq/L); ^†^symptoms presented.

We investigated whether the treatment recommendations, given by the European practice guidelines, ensure a well-controlled correction of hyponatremia.

## Subjects and methods

**Figure 1:**
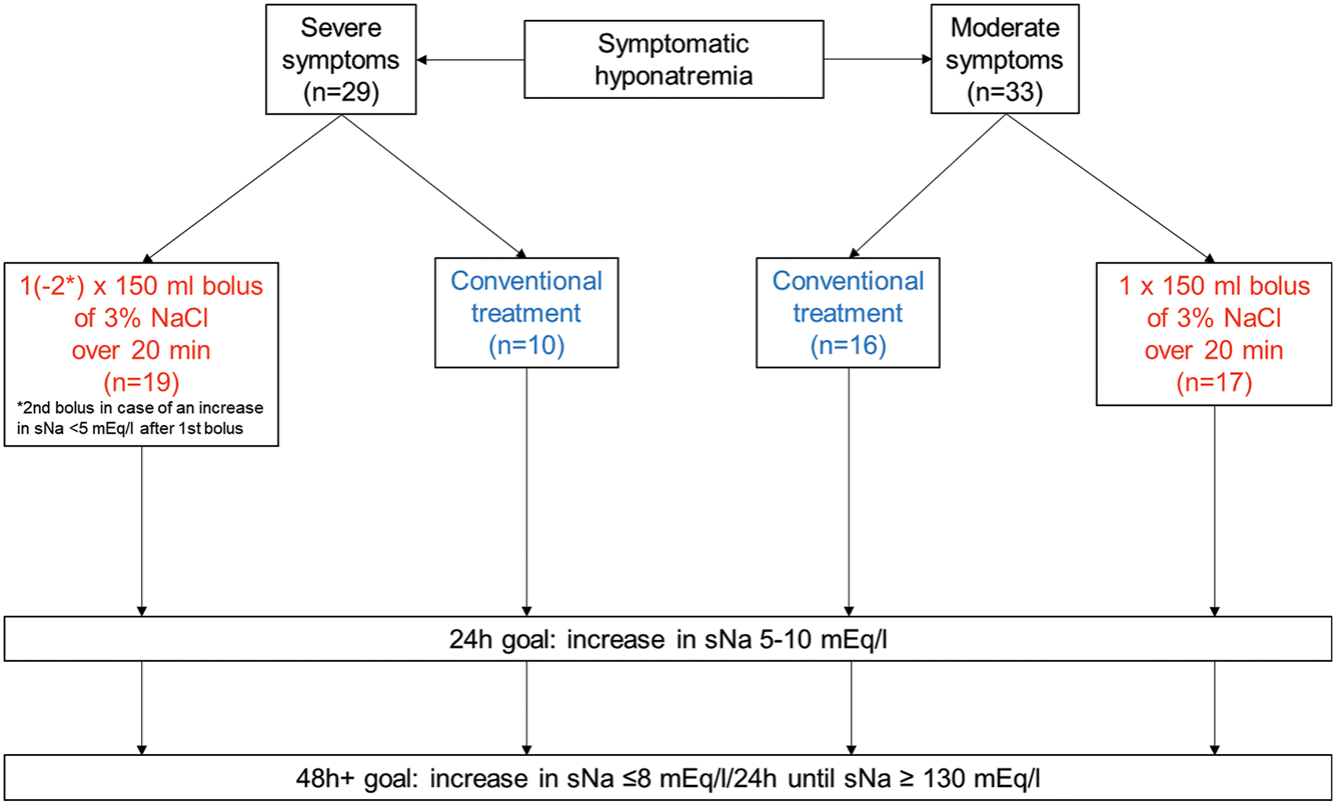
Flow-chart.

Between August 2015 and December 2017, a total of 72 patients with symptomatic HN who were admitted to the emergency department (ER) and intensive care unit (ICU) of the University Hospital of Wuerzburg were enrolled in this study. Patients with hyperosmolar HN (*n* = 2), patients treated with 5.85% saline solution (*n* = 5) and patients with incomplete charts (*n* = 3) were excluded from the final analysis. The remaining 62 patients were divided into groups according to (1) severity of clinical presentation (moderately or severely symptomatic) and (2) applied therapy, that is, administration of 3% NaCl (hypertonic saline group) or standard care (conventional treatment group) (Fig. 1). Moderate symptoms included headache, confusion or nausea, whereas emesis, cardiorespiratory distress, somnolence/coma (GCS ≤ 8) and seizures were classified as severe symptoms. The hypertonic saline group included patients receiving at least one bolus of 3% NaCl defined according to the European guidelines as 150 mL of 3% NaCl given over 20 min. Conventional treatment group included patients who received symptomatic and/or cause-specific treatment (e.g. isotonic saline or stopping HN-inducing medication).

Treating physicians were not influenced by the study in the decision-making process regarding the therapeutic approach. Emergency treatment with hypertonic saline was guided by the European guidelines, which were not implemented in a mandatory fashion but rather taught in daily practice by consultants and supported with posters in the ICU and ER. Briefly, in case of severe symptoms, the guidelines recommend a rapid increase in sNa by 5 mEq/L within 1 h after confirming HN by repeated administration of 150 mL boluses of 3% NaCl over 20 min each (Fig. 1). In case of moderate symptoms, the guidelines recommend a single 150 mL bolus of 3% NaCl over 20 min, aiming at an increase in serum sodium of at least 5 mEq/L over 24 h (Fig. 1).

The main outcome was to assess in accordance with the treatment goals in the first and every following 24 h after admission until sNa reached (or exceeded) 130 mEq/L or as far as sNa concentrations were documented. Treatment goals were defined as follows: increase in sNa by at least 5 mEq/L and maximum 10 mEq/L within the first 24 h after admission and limiting total increase in sNa to 8 mEq/L within each of the subsequent 24 h (Fig. 1). Overcorrection was defined as an increase in serum sodium >10 mEq/L at any time point during the first 24 h after admission and >8 mEq/L at any time point during each of the subsequent 24 h until sNa reached (or exceeded) 130 mEq/L. Sodium re-lowering after overcorrection was performed by administration of 5% glucose solution and/or desmopressin after consulting with an expert, as recommended by the European guidelines.

Magnitude of fluctuations in serum sodium by the coefficient of variation (CV), as previously described (10).

Data collection included the following parameters: demographics (age, sex, weight), symptoms (headache, vertigo, nausea, emesis, dyspnea, gait disorders, cognitive disorders, somnolence/coma), GCS (at admission, at 24 and at 48 h), symptom severity (moderate and severe), onset of HN (if known), underlying cause, medication, comorbidities, vital parameters at admission (systolic and diastolic blood pressure, heart rate, noninvasive pulse oximetry), volume status at admission (physical examination, symptoms and medication) and biochemical parameters (sNa at admission and every 4 h for the first 24 h followed by every 12 h until sNa reached or exceeded 130 mEq/L, first sNa after bolus administration, serum osmolality, serum potassium, serum glucose, serum creatinine, glomerular filtration rate (GFR), urine osmolality, urine sodium, urine potassium), urinary output within 24 and 48 h, incidence of osmotic demyelination syndrome, length of hospital stay and outcome (discharge or death in the hospital). In the hypertonic saline group, time from the first sodium measurement to the first and subsequent boluses of 3% NaCl and from each bolus of 3% NaCl to the subsequent sodium check were analyzed.

Collection of data were only performed after written informed consent. The study was approved by the local ethic committee at the university hospital of Wuerzburg (134/15).

Statistical analysis was performed using SPSS v. 24.0 and R project for statistical computing (version 3.5.3) (11). Differences in clinical and biochemical parameters were assessed using chi-square test, Student’s *t*-test, Mann–Whitney *U* test, and one-way ANOVA. Correlations were analyzed with Pearson’s or Spearman’s correlation test. Logistic regression models were applied to analyze the impact of patient parameters on the incidence of sodium overcorrection and were represented as odds ratios (OR) with 95% confidence intervals (95% CI). Relative risk (RR, 95% CI) for sodium overcorrection was evaluated separately for each subgroup. *P* values < 0.05 were considered to be statistically significant. Results were displayed as means ± s.d . or median and range, as appropriate.

## Results

### Description of the cohort

**Figure 2:**
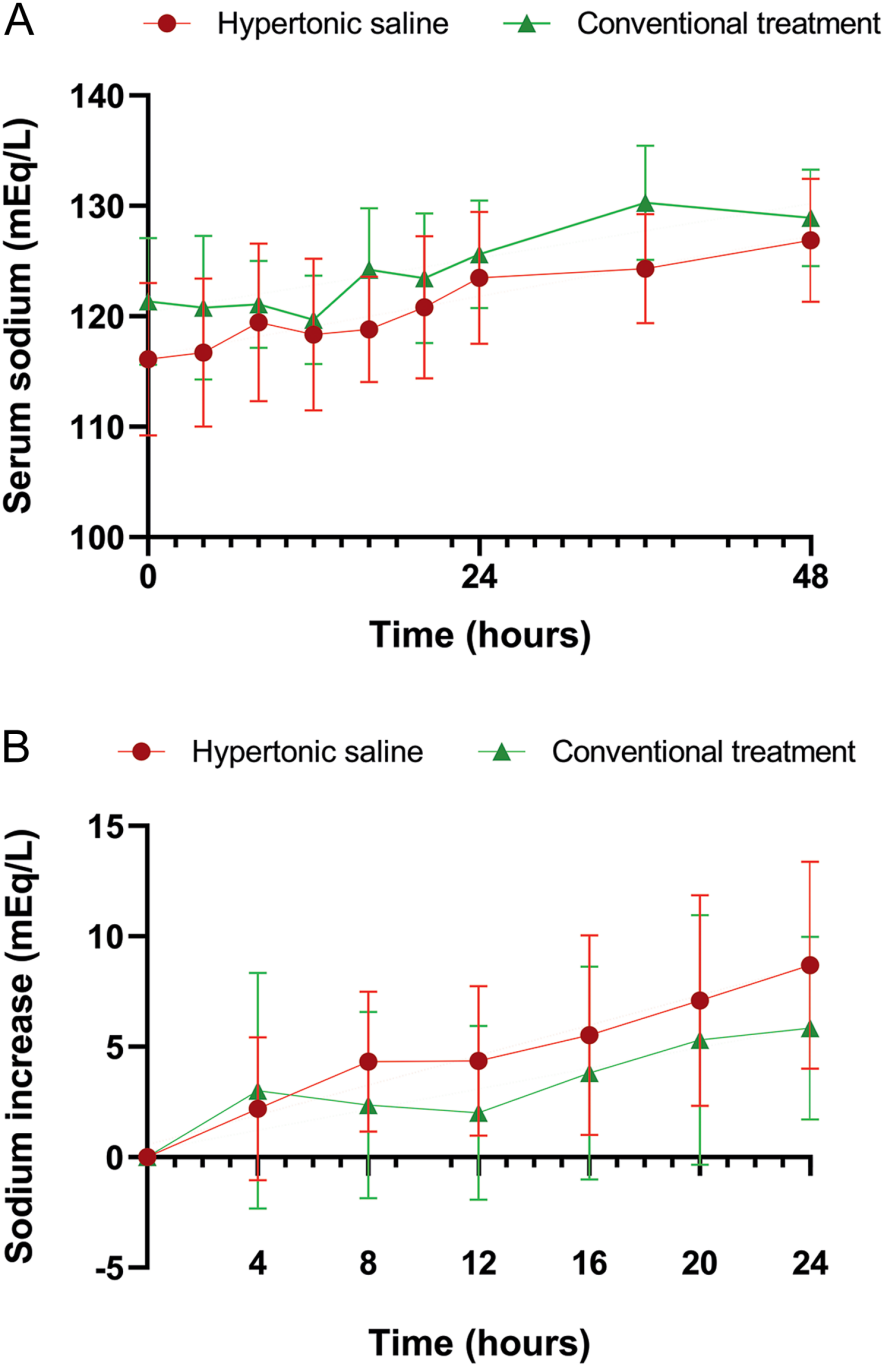
Changes in serum sodium concentration in hypertonic saline group vs conventional treatment group. (A) Changes in serum sodium concentration across the first 48 h after admission. (B) Sodium increase from baseline across the first 24 h after admission.

Baseline clinical and biochemical parameters of study participants are displayed in Table 2. Thirty-six patients (58%) received at least one bolus of 3% NaCl and 26 patients (42%) were treated conventionally. Thirty-three patients (53%) were moderately symptomatic and 29 patients (47%) were presented with severe symptoms. Vertigo was the most common manifestation of moderately symptomatic HN, whereas emesis and somnolence were the most prevalent initial presenting signs in severely symptomatic HN. sNa concentration at admission was significantly lower in the hypertonic saline group compared to conventional treatment group (116 ± 7 vs 121 ± 6 mEq/L, *P* **< 0.01) and in patients with severe symptoms compared to patients with moderate symptoms (115 ± 7 vs 121 ± 5 mEq/L, *P* **< 0.01) (Fig. 2 and Table 2).

**Table 2:** Baseline clinical and biochemical characteristics according to symptom, severity and treatment. Data are presented as *n* (%), mean ± s.d. or as median (min, max).

	Moderate symptoms	Severe symptoms	*P*	Conventional treatment group	Hypertonic saline group	*P*
Total *n*	33	29		26	36	
Male	14 (42)	12 (41)	NS	8 (31)	18 (50)	NS
Age	76 (23, 95)	66 (18, 90)	NS	66 (23, 95)	76 (18, 89)	NS
Weight, kg	69 ± 20	73 ± 12	NS	71 ± 22	71 ± 14	NS
Biochemical parameters						
sOsm, Osm/kg	254 (226, 301)	239 (107, 263)	<0.01	252 (222, 301)	242 (107, 263)	NS
sNa, mEq/L	121 ± 5	115 ± 7	<0.01	121 ± 6	116 ± 7	<0.01
sK, mEq/L	4.5 ± 0.8	4.3 ± 1.3	NS	4.5 ± 1.0	4.4 ± 1.0	NS
sGlc, mg/dL	122 (72, 275)	117 (51, 240)	NS	121 (89, 219)	118 (51, 275)	NS
GFR, mL/min/1.73qm	55 (4, 134)	90 (16, 375)	0.01	68 (13, 144)	84 (4, 375)	NS
Creatinine, mg/dL	1.3 (0.5, 10)	0.7 (0.3, 3)	0.021	1.2 (0.5, 3.6)	0.9 (0.3, 10)	NS
uOsm, mOsm/kg	286 (140, 610)	325 (90,709)	NS	286 (90, 554)	357 (128, 709)	NS
uNa, mEq/L	60 (20, 170)	38 (20, 177)	NS	49 (20, 89)	59 (20, 177)	NS
Symptoms						
Headache	9 (27)	6 (21)	NS	4 (15)	11 (31)	NS
Vertigo	21 (64)	12 (41)	NS	17 (65)	16 (44)	NS
Gait disorders	15 (45)	15 (52)	NS	13 (50)	17 (47)	NS
Cognitive disorders	15 (45)	19 (66)	0.012	10 (38)	24 (67)	0.021
Nausea	14 (42)	14 (48)	NS	10 (38)	18 (50)	NS
Emesis	3 (9)^*^	13 (45)	<0.01	5 (19)	11 (31)	NS
Dyspnea	11 (33)^*^	4 (14)	NS	7 (27)	8 (22)	NS
Somnolence/coma	1 (3)	9 (31)	<0.01	3 (12)	7 (19)	NS
Systolic BP, mmHg	140 (71, 200)	140 (93,180)	NS	139 (94, 200)	140 (71, 200)	NS
Diastolic BP, mmHg	70 (43, 111)	63 (37, 105)	NS	70 (46, 100)	68 (37, 111)	NS
Heart rate, bpm	76 ± 13	82 ± 20	NS	75 ± 13	81 ± 18	NS
Volume status						
Hypovolemic	12 (36)	17 (59)	NS	13 (50)	16 (44)	NS
Euovolemic	13 (40)	10 (35)	NS	9 (35)	14 (39)	NS
Hypervolemic	7 (21)	1 (3)	NS	2 (8)	6 (17)	NS
n.a.	1 (3)	1 (3)	NS	2 (8)	0 (0)	NS
GCS						
Admission	15 (15, 15)	15 (3,15)	NS	15 (3, 15)	15 (10, 15)	NS
0–24 h	15 (15, 15)	15 (14,15)	NS	15 (15, 15)	15 (14, 15)	NS
24–48 h	15 (15, 15)	15 (14,15)	NS	15 (15, 15)	15 (14, 15)	NS

*Due to underlying condition leading to hyponatremia.

GFR, glomerular filtration rate; n.a., not assessed; sGlc, serum glucose; sK, serum potassium; sNa, serum sodium; sOsm, serum osmolality; uNa, spot urine sodium; uOsm, spot urine osmolality.

The most frequent etiology of HN was renal sodium loss due to thiazide-type diuretics (30%) followed by acute infections of the respiratory (15%) and gastrointestinal tract (11%) (Supplementary Table 1, see section on supplementary materials given at the end of this article). Pre-existing conditions and medication are displayed in Supplementary Table 2. Data regarding the onset of HN (proven acute and proven chronic) could only be assessed in 21% (*n* = 13) of patients. Thus, the majority was classified as (unproven) chronic due to lack of sodium measurements prior to admission.

### Increase in serum sodium and risk of overcorrection

The mean increase in sNa after 24 and 48 h was within the recommended range both in the whole cohort (8 ± 5 mEq/L and 11 ± 9 mEq/L, respectively) and in each of the analyzed subgroup as shown in Table 3. The median CV of changes in sNa assessed every 4 h during the first 24 h after admission was 1.35%. Significantly more patients from the conventional treatment group had a CV ≥ 1.35% compared to patients from the hypertonic saline group (87.5% vs 36.4%, *P *= 0.013) and were thus exposed to higher degrees of fluctuations in sNa concentrations (Fig. 2).

**Table 3:** Mean increase in serum sodium (sNa) at 24 and 48 h after admission according to treatment and symptom severity.

	*n*	Mean change in serum sodium (mEq/L)		Mean serum sodium (mEq/L)		
		At 24 h	At 48 h	Baseline	At 24 h	At 48 h
Whole cohort	62	8 ± 5	11 ± 9	118 ± 7^†‡^	124 ± 6^§^	128 ± 5
Hypertonic saline group	36	9 ± 5	12 ± 9	116 ± 7^†‡*^	123 ± 6^§^	127 ± 6
Conventional treatment group	26	6 ± 4	9 ± 8	121 ± 6^†‡^	126 ± 5	129 ± 4
Moderate symptoms	33	5 ± 4^**^	7 ± 7^**^	121 ± 5^†‡^	125 ± 4	127 ± 5
Severe symptoms	29	10 ± 4	14 ± 9	115 ± 7^†‡^	123 ± 7^§^	128 ± 5
sNa at admission, mEq/L						
<120	30	10 ± 4^***^	16 ± 7^***^	113 ± 5^†‡^	121 ± 5^§^	127 ± 5
≥120	32	5 ± 4	5 ± 6	124 ± 3^†‡^	128 ± 4	128 ± 5

^†^*P* < 0.05 baseline compared to 24h; ^‡^*P* < 0.05 baseline compared to 48 h; ^§^*P* < 0.05 24 h compared to 48 h; *P < 0.05 hypertonic saline group compared to conventional treatment group; **P < 0.05 moderate symptoms compared to severe symptoms; ***P < 0.05 admission sNa < 120 mEq/L compared to admission sNa ≥ 120 mEq/L.

**Figure 3:**
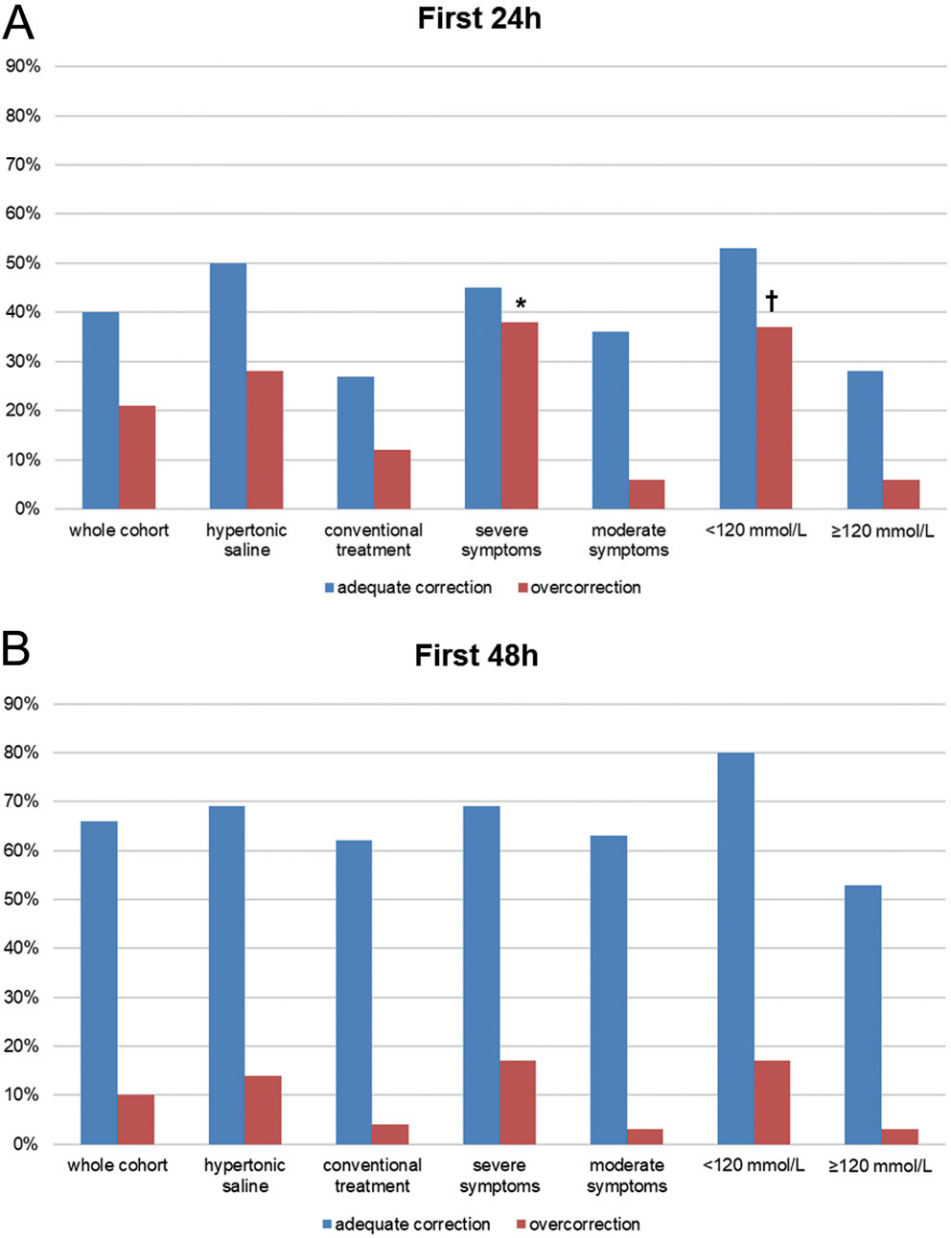
Proportion of adequate correction and overcorrection of serum sodium over (A) 24 h from admission and (B) 48 h from admission in the whole cohort and in patient subgroups according to treatment, symptom severity and initial serum sodium. Fisher’s exact test, *P < 0.05 compared to moderate symptoms, ^†^*P* < 0.05 compared to serum sodium ≥ 120 mEq/L at admission.

Highest incidence of overcorrection was registered during the first 24 h of therapy and occurred more often in patients treated with 3% NaCl, in severely symptomatic patients and in patients with a baseline sNa < 120 mEq/L in comparison to corresponding control groups (Fig. 3 and Supplementary Table 3). Compared with conventional strategies, the relative risk for overly rapid correction at any time point during the first 24 h was 2.4 (95% CI: 0.7–7.9) for patients treated with 3% NaCl. Highest risk for overcorrection during the first 24 h among patients treated with 3% NaCl was registered for severely symptomatic patients (RR: 8.0, 95% CI: 1.6–47, *P *< 0.01 compared to moderately symptomatic patients), followed by patients with a baseline sNa < 120 mEq/L (RR: 5.0, 95% CI: 1.0–29.9, *P * = 0.04 compared to patients with a baseline sNa ≥ 120 mEq/L). Patients with severely symptomatic HN received a second hypertonic infusion more often compared with moderately symptomatic patients (7/19 vs 2/17, *P * = 0.08).

Urine output was documented in 45% of the patients. Diuresis was correlated positively with the increase in sNa in the whole cohort (r = 0.6, *P* < 0.01) as well as in the bolus group (r = 0.6, *P *< 0.01). Diuresis was significantly higher in patients experiencing overcorrection (>10 mEq/L) (4000 (870–7080) mL vs 1500 (0–4700) mL, *P *= 0.02) (Supplementary Table 4), which was also confirmed after excluding patients treated with loop diuretics (*n* = 5). No difference was found regarding treatment strategy. Clinically assessed volume status at admission, however, had no significant impact on the overcorrection.

In ten (28%) patients from the bolus group, sodium was relowered with 5% glucose (*n* = 3), desmopressin (*n* = 2) or both (*n* = 5). Median time from first bolus to relowering was 7 (0–18) h. Overcorrection was prevented in four (40%) patients. Patients needing relowering had a significantly lower baseline sNa and registered a significantly higher increase in sNa after the first bolus compared to patients without relowering (111 ± 8 mEq/L vs 118 ± 6 mEq/L *P *< 0.01 and 5 ± 4 mEq/L vs 2 ± 3 mEq/L *P *= 0.02).

For the whole cohort, a logistic regression analysis comprising symptom severity, type of therapy and sNa at admission confirmed symptom severity to be an independent prognostic parameter for overcorrection during the first 24 h (OR: 6.6, 95% CI: 1.2–35.3, *P *= 0.028). Also, for patients treated with 3% NaCl, symptom severity was the only independent prognostic factor for overcorrection in the first 24 h (OR: 14.1, 95% CI: 1.2–163.9, *P* = 0.034) according to a logistic regression model also including time to first bolus and first reported sodium change after administration of 3% NaCl.

The degree of excessive sodium correction in the first 24 h did not differ between hypertonic saline and conventional treatment group (mean excessive increase from baseline 14 ± 2 vs 13 ± 2 mEq/L, *P *= 0.6) nor between severe and moderately symptomatic patients regardless of treatment strategy (mean excessive increase from baseline 14 ± 2 vs 14 ± 3 mEq/L, *P *= 0.9).

Baseline sNa negatively correlated with changes in sNa across the first 48 h after admission both in the whole cohort (0–24 h: r = −0.7, *P *< 0.01, 24–48 h: r = −0.7, *P *< 0.01, 0–48 h: r = −0.8, *P *< 0.01) and in each of the treatment subgroups (conventional: 0–24 h: r = −0.7, *P *= 0.01, 0–48 h: r = −0.8, *P *< 0.01, hypertonic saline: 0–24 h: r = −0.6, *P *< 0.01, 24–48 h: r = −0.7, *P *< 0.01, 0–48 h: r = −0.8, *P *< 0.01).

### Risk of insufficient correction

During the first 24 h, sNa failed to increase by at least 5 mEq/L in 38% of all patients. Patients most exposed to an insufficient increase in sNa were those receiving conventional treatment (62% vs 22% compared to hypertonic saline group, *P *= 0.03, RR: 2.8, 95% CI: 1.4–5.5), moderately symptomatic patients (58% vs 17% compared to severely symptomatic patients, *P *< 0.01, RR: 2.8, 95% CI: 1.4–5.5) and patients with an initial sNa ≥ 120 mEq/L (66% vs 10% compared to patients with an initial sNa < 120 mEq/L, *P *< 0.01, RR: 2.6, 95% CI: 1.6–4.3).

Of the 36 bolus-patients, 28% (*n* = 10) achieved the 5 mEq/L goal after the first bolus (6.5 ± 0.8 mEq/L), 53% (*n* = 19) registered an increase in sNa < 5 mEq/L (2.3 ± 0.2 mEq/L), whereas 19% (*n* = 7) registered either no increase (*n* = 3) or a further drop in sNa (*n* = 4, −1.6 ± 1.6 mEq/L) (non-responders). Among non-responders, only two received a second bolus. Non-responders had a median baseline sNa > 120 mEq/L.

### Clinical outcome

In total, 89% of the patients were discharged and 11% died in hospital due to complications from underlying diseases other than hyponatremia. Length of hospital stay did not significantly differ between subgroups or between patients with or without overcorrection (Supplementary Table 5). Osmotic demyelination syndrome was suspected in one patient who was presented with profound hyponatremia (115 mEq/L) and who was newly diagnosed with secondary adrenal insufficiency on the sixth day after admission. The patient recovered completely and the MRI was canceled.

## Discussion

Our study was designed to evaluate the safety of hypertonic saline bolus administration for treatment of symptomatic HN, as recommended by the European practice guidelines (7). This ‘real world study’ provides the following key conclusions: with hypertonic bolus of saline, sNa increased in a steady fashion in the first 24 h, while conventional treatment led to more pronounced fluctuations and increased the risk of an insufficient rise in sNa, indicating that this approach alone can be harmful, as previously reported by Greenberg *et al*. (1). Another important finding is that overcorrection is frequent in clinical routine and appeared in 21% of all patients but was highest in patients with severe symptoms treated with hypertonic saline (9 of 19 patients, 47%).

Severely symptomatic patients received a second hypertonic saline bolus more often and were at the same time at the highest risk for overcorrection. This finding is in contrast to the European practice guidelines (7), which always recommend a second bolus in severely symptomatic patients to achieve a combined first-hour goal of (1) relief of symptoms and (2) increase in sNa of at least 5 mEq/L. In our cohort, a second bolus was usually administered not immediately but later within the first 24–48 h in case of an insufficient biochemical response to the first bolus in severely symptomatic patients.

However, also patients treated with a single bolus of hypertonic saline were prone to overcorrection. Interestingly, the bolus approach was recommended by two guidelines independently but with subtle differences. The European guidelines defined a bolus as 150 mL of 3% NaCl, while the American recommendations defined a bolus as 100 mL of 3% NaCl (8, 12). The European guidelines recommended at minimum a total of two boluses in severely symptomatic patients and the American recommendations allowed up to three boluses, ending up with the same amount of fluid and sodium in selected cases. However, the reduced dose in volume and saline of the American recommendations could result in a more controlled increase in sNa and, therefore, could be superior in terms of safety (13). Comparing hypertonic bolus administration with a continuous infusion of hypertonic saline in patients with symptomatic HN due to SIADH, Garrahy *et al.* (14) also used the 100 mL dose bolus and found that bolus administration was superior to the continuous infusion of hypertonic saline in terms of recommended short-term sodium elevation (4 to 6 mEq/L over 6 h) to reverse neurologic symptoms of cerebral edema. However, bolus administration required more often use of dextrose and DDAVP to prevent overcorrection in the first 24 h, especially in patients receiving a total of three boluses. The recently published prospective multicentric randomized SALSA trial compared weight adjusted hypertonic saline bolus with a slow continuous hypertonic saline infusion in symptomatic hyponatremia. This study found that hypertonic boluses were superior in achieving the targeted increase in sNa without exposing patients to a higher overcorrection rates (15). Bolus therapy required less relowering interventions compared to the continuous infusion (15).

Therefore, reducing both the bolus volume and the total volume of administered hypertonic saline might be necessary to avoid overcorrection in the critical first 24 h.

However, symptom severity might have been overrated in some cases, since somnolence may have also been caused by dehydration rather than HN-induced cerebral pressure. Therefore, special attention should be paid to understand whether HN or hypovolemia is the cause of impaired mental status, since misinterpretation may result in overdiagnosis of severely symptomatic HN. Indeed, hypovolemic patients were classified more often as having severe than moderate symptomatic hyponatremia (68% vs 37%, *P *= 0.045).

Another known risk factor for overcorrection is increasing diuresis as a result of antagonizing ADH-mediated free water retention by volume repletion or simply discontinuing HN-inducing drugs, especially in hypovolemic patients (16, 17, 18, 19). Urine output was documented only in 45% of our patients. With this limitation, we still found that a higher urine output was significantly correlated with overcorrection and diuresis was therefore identified as a risk factor for overcorrection.

Special consideration should also be given to patients with profound HN, as these are more prone to overly rapid correction (16, 17, 20, 21). Our study provides new evidence that a sNa < 120 mEq/L is an independent risk factor for overcorrection regardless of symptom severity and requires a more cautious approach focusing mainly on improving symptoms rather than achieving predefined goals. A retrospective observational study conducted in Japan in 56 patients with HN revealed that for each decrease in in pretreatment sNa of 4 mEq/L, the risk of overly rapid correction was doubled (17). Mohmand *et al.* analyzed not only the adherence to recommended correction limits (<12 mEq/L per 24 h and <18 mEq/L per 48 h) but also the accuracy of predicting the increase in sNa using the Adrogué-Madias formula in 62 hyponatremic patients treated with 3% hypertonic saline (16, 22). Overcorrection only occurred in patients with an initial sNa < 120 mEq/L and exceeded by 2.4 times the expected increase in sNa despite being lower than the recommended infusion rates of hypertonic saline. Limiting sodium increase to 8 mEq/L in the first 24 h and 6 mEq/L in the subsequent 24 h – as already suggested for patients with an initial sNa < 106 mEq/L (20) – might thus be a safe and feasible strategy to apply in all patients with sNa < 120 mEq/L to avoid unpredictable overcorrection.

Sodium failed to increase after the first bolus of 3% NaCl in 19% of our patients. No recommendations are available in the guidelines to treat non-responders. The reason for non-responding is unclear, and the limited number of observations preclude sound explanations. To speculate, however, in hypovolemic patients with preserved diuresis, excess sodium may allow a further increase in renal fluid retention – resulting in a (moderate) drop of sNa. Indeed, diuresis in non-responders was not different to responders in our study. Since bolus administration was overall associated with a more controlled correction of hyponatremia, it should not be withheld in hypovolemic patients and volume administration, in contrast, may result in a very uncontrolled sodium rise.

Moreover, no clinical deterioration was documented for this subgroup of non-responders. Therefore, we believe that it may be better not to recommend an immediate second bolus of 150 mL 3%NaCl but instead rely on clinical presentation and decide about treatment intensification sometimes later. Neurologic outcome was also not different in the trial from Garrahy *et al.* and in the SALSA trial although sodium increased faster in the bolus groups of both trials (14, 15).

Picturing clinical routine is a strength of this study and also its greatest limitation. No randomization was performed, and treatment decisions were left upon the discretion of the treating physicians in the ICU and ER. In support of our findings, similar results were also reported by the hyponatremia registry conducted both in Europe and in the United States and points once again toward the pitfalls of a rather easy to follow diagnostic work-up. Furthermore, data on volume balance are missing and evaluation on day 5–10 is required in future studies to determine the incidence of osmotic demyelination. However, our study indicates that a refined bolus-based treatment strategy may achieve the critical level of feasibility to become a reliable clinical treatment strategy in the future.

In conclusion, our study did not show an improvement in safety regarding overcorrection with a bolus-based treatment strategy. However, we found that the advantage of (1) a symptom-triggered treatment decision with (2) a bolus-based treatment algorithm is significant and therefore, refining the bolus strategy is worthwhile. We hypothesized that cautiously waiting before repeating the administration of hypertonic saline and reducing the volume of hypertonic saline bolus should be taken into consideration.

## Supplementary Material

Supplementary Table 1. Underlying cause of hyponatremia

Supplementary Table 2. Pre-existing conditions and medication according to symptom severity and treatment

Supplementary Table 3. Correction of serum sodium within recommended range and overcorrection rate at 24 and 48 hours after admission according to treatment, symptom severity and serum sodium at admission

Supplementary Table 4. Diuresis (ml, median (min, max)) during the first 24 hours after admission according to treatment, symptom severity and overcorrection status.

Supplementary Table 5. Length of hospital stay (days, median (min, max)) according to treatment, symptom severity and overcorrection status at 24 h.

## Declaration of interest

M F is an associate editor for the European Journal of Endocrinology. However, he was not involved in any way in the review or editorial process for this paper on which he is listed as authors. The other authors have nothing to disclose.

## Funding

This work was supported by the Deutsche Forschungsgemeinschaft (DFG) within the CRC/Transregio 205/1 ‘The Adrenal: Central Relay in Health and Disease’ and the Clinician Scientist Program ‘Rare Important Syndromes in Endocrinology’ (RISE).
