# Supplementary material for: Treatment of symptomatic hyponatremia with hypertonic saline: a real-life observational study
Source: Eur J Endocrinol. 2021 Feb 25;184(5):647–55. doi: 10.1530/EJE-20-1207 (PMC8052513; doi:10.1530/EJE-20-1207)
Supplement: Supplementary Table 1. Underlying cause of hyponatremia [file supplementary_table_1.pdf]

Supplementary Table 1. Underlying cause of hyponatremia

Underlying cause	n (%)
Diuretic therapy	19 (31%)
Thiazide-type diuretic	18 (30%)
Loop diuretics	3 (5%)
Acute infection	21 (34%)
Respiratory tract	9 (15%)
Gastrointestinal	7 (11%)
Urinary tract	3 (5%)
Unknown etiology	4 (6%)
Acute decompensated heart failure	8 (13%)
Adrenal insufficiency	8 (13%)
SIADH	7 (11%)
Unknown origin	3 (5%)
Water intoxication due to desmopressin overdose	2 (3%)
Alcohol intoxication	2 (3%)
Acute liver failure	1 (2%)
Acute renal failure	1 (2%)
Chemotherapy	1 (2%)
Psychotropic drugs	1 (2%)
Recreational drugs	1 (2%)
Water intoxication due to primary polydipsia	1 (2%)
