# Supplementary material for: Treatment of symptomatic hyponatremia with hypertonic saline: a real-life observational study
Source: Eur J Endocrinol. 2021 Feb 25;184(5):647–55. doi: 10.1530/EJE-20-1207 (PMC8052513; doi:10.1530/EJE-20-1207)
Supplement: Supplementary Table 2. Pre-existing conditions and medication according to symptom severity and treatment [file supplementary_table_2.pdf]

Supplementary Table 2. Pre-existing conditions and medication according to symptom severity and treatment

	Moderate symptoms (n=33)	Severe symptoms (n=29)	p	Conventional treatment group (n=26)	Hypertonic saline group (n=36)	p
Pre-existing conditions, n (%)						
Diabetes mellitus	4 (12)	4 (14)	NS	3 (12)	5 (14)	NS
Diabetes insipidus	0 (0)	1 (3)	NS	0 (0)	1 (3)	NS
Chronic kidney disease	17 (52)	9 (31)	NS	12 (46)	14 (39)	NS
Hepatopathy	3 (9)	5 (17)	NS	2 (8)	6 (17)	NS
Heart failure	7 (21)	4 (14)	NS	5 (19)	6 (17)	NS
Adrenal insufficiency	4 (12)	3 (10)	NS	4 (15)	3 (8)	NS
Psychiatric disorders	4 (12)	6 (21)	NS	7 (27)	3 (8)	NS
SIADH	1 (3)	1 (3)	NS	1 (4)	1 (3)	NS
Medication, n (%)						
Psychiatric medication	10 (30)	4 (14)	NS	8 (31)	6 (17)	NS
Diuretics	20 (61)	12 (41)	NS	12 (46)	20 (56)	NS
Analgesics	5 (15)	7 (24)	NS	6 (23)	6 (17)	NS
Glucocorticoids	6 (18)	3 (10)	NS	3 (12)	6 (17)	NS
Mineralocorticoids	1 (3)	0 (0)	NS	1 (4)	0 (0)	NS
Chemotherapy	2 (6)	3 (10)	NS	2 (8)	3 (8)	NS
Vasopressin analogues	0 (0)	2 (7)	NS	0 (0)	2 (6)	NS
