# Supplementary material for: Treatment of symptomatic hyponatremia with hypertonic saline: a real-life observational study
Source: Eur J Endocrinol. 2021 Feb 25;184(5):647–55. doi: 10.1530/EJE-20-1207 (PMC8052513; doi:10.1530/EJE-20-1207)
Supplement: Supplementary Table 3. Correction of serum sodium within recommended range and overcorrection rate at 24 and 48 hours after admission according to treatment, symptom severity and serum sodium at admission [file supplementary_table_3.pdf]

Supplementary Table 3. Correction of serum sodium within recommended range and overcorrection rate at 24 and 48 hours after admission according to treatment, symptom severity and serum sodium at admission

	Correction of serum sodium within recommended range (%)		Overcorrection rate (%)	
Patients	At 24h	At 48h	At 24h	At 48h
Whole cohort (n=62)	40	66	21	10
Hypertonic saline bolus group (n=36)	50	69	28	14
Conventional treatment group (n=26)	27	62	12	4
Severe symptoms (n=29)	36	63	38*	17
Moderate symptoms (n=33)	45	69	6	3
sNa < 120 mEq/L at admission (n=30)	53	80†	37†	17
sNa ≥ 120 mEq/L at admission (n=32)	28	53	6	3

* $p<0.05$ compared to moderate symptoms

† $p<0.05$ compared to sNa ≥ 120 mEq/L at admission
