# Supplementary material for: Treatment of symptomatic hyponatremia with hypertonic saline: a real-life observational study
Source: Eur J Endocrinol. 2021 Feb 25;184(5):647–55. doi: 10.1530/EJE-20-1207 (PMC8052513; doi:10.1530/EJE-20-1207)
Supplement: Supplementary Table 4. Diuresis (ml, median (min, max)) during the first 24 hours after admission according to treatment, symptom severity and overcorrection status. [file supplementary_table_4.pdf]

Supplementary Table 4. Diuresis (ml, median (min, max)) during the first 24 hours after admission according to treatment, symptom severity and overcorrection status.

Symptom severity			Therapy group			Overcorrection		
moderate	severe	p	CT	HS	p	no	yes	p
2000<br>(0-4700)	2700<br>(450-7080)	NS	1550<br>(0-4700)	3530<br>(450-7080)	NS	1500<br>(0-4700)	4000<br>(870-7080)	0.02

*CT: conventional treatment, HS: hypertonic saline
