# Supplementary material for: Treatment of symptomatic hyponatremia with hypertonic saline: a real-life observational study
Source: Eur J Endocrinol. 2021 Feb 25;184(5):647–55. doi: 10.1530/EJE-20-1207 (PMC8052513; doi:10.1530/EJE-20-1207)
Supplement: Supplementary Table 5. Length of hospital stay (days, median (min, max)) according to treatment, symptom severity and overcorrection status at 24 h. [file supplementary_table_5.pdf]

Supplementary Table 5. Length of hospital stay (days, median (min, max)) according to treatment, symptom severity and overcorrection status at 24 h.

Symptom severity			Therapy group			Overcorrection		
moderate	severe	p	CT	HS	p	no	yes	p
9 (2-196)	11 (2-136)	NS	8 (2-196)	11 (2-136)	NS	9 (2-196)	11 (5-41)	NS

*CT: conventional treatment, HS: hypertonic saline
